# Study of the heavily p-type doping of cubic GaN with Mg

**DOI:** 10.1038/s41598-020-73872-w

**Published:** 2020-10-08

**Authors:** C. A. Hernández-Gutiérrez, Y. L. Casallas-Moreno, Victor-Tapio Rangel-Kuoppa, Dagoberto Cardona, Yaoqiao Hu, Yuri Kudriatsev, M. A. Zambrano-Serrano, S. Gallardo-Hernandez, M. Lopez-Lopez

**Affiliations:** 1 Tecnológico Nacional de México/Instituto Tecnológico de Tuxtla Gutiérrez, Posgrado en Ingeniería Grupo de Opto-mecatrónica, Carretera Panamericana km 1080, 29050 Tuxtla Gutiérrez, Mexico; 2 CONACYT, Instituto Politécnico Nacional - UPIITA, Av. IPN 2580 Col. Barrio La Laguna Ticomán, Ciudad de México, 07340 Mexico; 3 Centro de Investigación en Dispositivos Semiconductores, Instituto de Ciencias, Universidad Autónoma de Puebla, Puebla, 72000 Mexico; 4 Facultad de Ciencias Físico-Matemáticas, UMSNH, Edificio L, Francisco J. Mujica S/N, Morelia, Michoacán 58000 Mexico; 5 Department of Materials Science and Engineering, The University of Texas At Dallas, Richardson, TX 75080 USA; 6 Departamento de Ingeniería Eléctrica SEES, Cinvestav-IPN, Ciudad de México, 07360 Mexico; 7 Programa de Doctorado en Nanociencias Y Nanotecnología, Cinvestav-IPN, Ciudad de México, 07360 Mexico; 8 Departamento de Física, Cinvestav-IPN, Ciudad de México, 07360 Mexico

**Keywords:** Materials science, Materials for devices, Electronic devices

## Abstract

We have studied the Mg doping of cubic GaN grown by plasma-assisted Molecular Beam Epitaxy (PA-MBE) over GaAs (001) substrates. In particular, we concentrated on conditions to obtain heavy p-type doping to achieve low resistance films which can be used in bipolar devices. We simulated the Mg-doped GaN transport properties by density functional theory (DFT) to compare with the experimental data. Mg-doped GaN cubic epitaxial layers grown under optimized conditions show a free hole carrier concentration with a maximum value of 6 × 10^19^ cm^−3^ and mobility of 3 cm^2^/Vs. Deep level transient spectroscopy shows the presence of a trap with an activation energy of 114 meV presumably associated with nitrogen vacancies, which could be the cause for the observed self-compensation behavior in heavily Mg-doped GaN involving Mg-V_N_ complexes. Furthermore, valence band analysis by X-ray photoelectron spectroscopy and photoluminescence spectroscopy revealed an Mg ionization energy of about 100 meV, which agrees quite well with the value of 99.6 meV obtained by DFT. Our results show that the cubic phase is a suitable alternative to generate a high free hole carrier concentration for GaN.

## Introduction

Gallium Nitride (GaN) in the wurtzite or hexagonal phase (h-GaN) has been intensively studied during the last decades for its unique properties. It has a large direct band gap with high saturation velocity, which makes it a promising candidate to achieve high power devices^1–4^. However, spontaneous piezoelectric polarization occurs in h-GaN inducing internal electric fields, which causes energy band tilting^5^. These internal electric fields significantly influence optoelectronic devices' performance^6^. This is not the case for the much less explored zincblende or cubic phase of GaN (c-GaN). Due to the crystal symmetry of c-GaN, spontaneous piezoelectric polarization can be avoided, making it a more suitable material for the described applications^7^. At the same time, c-GaN offers additional advantages like; greater hole mobility and a smaller band gap of 3.2 eV at room temperature (RT) compared with 3.4 eV of h-GaN. This last characteristic is an extra advantage when alloying c-GaN with c-InN (with a bandgap of 0.66 eV at RT), as less In is required to achieve smaller band gaps^8,9^. Regarding optoelectronic applications, there is a wider choice of cubic substrates to grow c-GaN, such as GaAs and SiC^10,11^. Growing on these cubic substrates allows easier cleavage, enabling mirror-like edges suitable for laser applications. At the same time, the (001) oriented substrates induce natural stacking fault annihilation, as they propagate along 60° orientations^11^. Moreover, employing Si- and/or GaAs-based substrates paves the way for the use of the well-developed device processing technology on these substrates. Due to these advantages, nitrides in the cubic phase are strong candidates to develop semiconductor devices such as LEDs and photovoltaic devices. However, c-GaN is metastable, causing its growth a difficult challenge^12^. An additional drawback is that under typical growth conditions hexagonal GaN has an intrinsic n-type nature, and if GaN is aimed for optoelectronic applications, then the development of p-type doping of GaN is fundamental for any p–n junction-based device. To achieve p-type characteristics in h-GaN, Mg doping was proposed^13^. However, for Mg-doped h-GaN there are major issues, namely: (a) a large acceptor activation energy ≃ 200–265 meV, (b) Mg solubility limit around 10^20^ cm^−3^, and (c) compensation by native defects^14,15^.

On the other hand, for c-GaN, theoretical calculations have predicted ionization energies for Mg acceptors around 130 meV^16^, in contrast with experimental reports about 165 meV^17^ and 230 meV^18^. Despite these early studies have been done almost three decades ago, the mentioned metastability nature of c-GaN and the Mg doping drawbacks have caused disperse and limited information on the electrical properties of Mg-doped cubic GaN^19–24^. Recently the authors in reference^25^ reported a free hole concentration in the order of 10^19^ cm^−3^ in c-GaN. No subsequent study could be found where hole concentration is in the order of 10^19^ cm^−3^ or larger. Thus, further research is necessary to achieve a better understanding of the Mg doping of c-GaN. This paper intends to report a comprehensive study of cubic GaN doped with Mg, employing density functional theory (DFT) supported with the experimental techniques of x-ray photoelectron spectroscopy valence band maximum (XPS-VBM), deep level transient spectroscopy (DLTS), and photoluminescence spectroscopy (PL). With special emphasis on demonstrating that cubic GaN is suitable to achieve a high free hole concentration when doped with Mg under appropriate conditions. We extracted the Mg activation energy by two different techniques obtaining results that are quite close to the theoretical prediction.

### DFT simulation

First-principles simulation was performed to gain insight into the hole mobility and Mg ionization energy level in cubic GaN. The simulation was conducted by DFT as implemented by VASP^26^ using projected augmented wave (PAW)^27,28^ pseudopotentials. Perdew-Burke-Ernzerhof generalized gradient approximation (GGA-PBE) functional was employed to depict the exchange–correlation potential energy. For all calculations, an energy cutoff of 520 eV was adopted for plane wave basis expansion. Brillouin-zone integrations were performed based on the Gamma-centred Monkhorst–Pack *k*-point mesh, with a sampling density of 0.03 Å^−1^. Structures were relaxed using the conjugate gradient (CG) method with the convergence criterion of the force on each atom less than 0.02 eV/Å. The converged energy criterion is 10^–5^ eV for electronic minimization. The mobility μ depends on the effective mass m* through μ = eτ/m* where the relaxation time τ depends on different scattering mechanisms, and the effective mass was calculated according to the band structure dispersion relationship: ${m}^{*}={\hslash }^{2}/\frac{{d}^{2}E}{{d}k^{2}}$. Herein we took into account both phonons scattering and ionized impurity scattering. We computed scattering rates at a temperature of 300 K. Calculation details and procedure can be found elsewhere^29^.

The point defect computations were performed using the supercell technique adopting 2 × 2 × 2 supercells of the primitive cell to mimic the dilute doping condition while ensuring a reasonable computation cost. The defect states with the charge *q* were corrected using the Freysoldt scheme^30^, as implemented in the PyCDT package^31^. The Freysoldt scheme leads to the calculated formation energies approaching to the dilute-defect limit and independent of the supercell size^32^. The formation energy of defect *D* in charged state *q E*^*f*^[*D*^*q*^] can be written as^33^

$$\it {\text{E}}^{\rm f}\left[{\text{D}}^{\rm q}\right] = {\text{E}}_{\rm def}\left[{\text{D}}^{\rm q}\right] - {\text{E}}_{\rm bulk} - \sum {\text{n}}_{\rm i}{\mu}_{\rm i}+ \text{q} ({\text{E}}_{\rm F} +{\text{E}}_{\rm VBM})+{\text{E}}_{\rm corr}\left[{\text{D}}^{\rm q}\right]$$

where *E*_def_[*D*^*q*^] is the energy of the supercell with defects, *E*_bulk_ is the energy of the perfect supercell without any defects, *n*_*i*_ indicates the number of the *i*th-atoms that have been added into (*n*_*i*_ > 0) or removed from (*n*_*i*_ < 0) the supercell and *μ*_*i*_ is the chemical potential of defective atoms. *E*_VBM_ is the energy of the valence band maximum (VBM), and *E*_F_ is the Fermi level referenced to *E*_VBM_. The correction term *E*_corr_[*D*^*q*^] is introduced to take into account the spurious interactions between the charges and their images.

**Figure 1:**
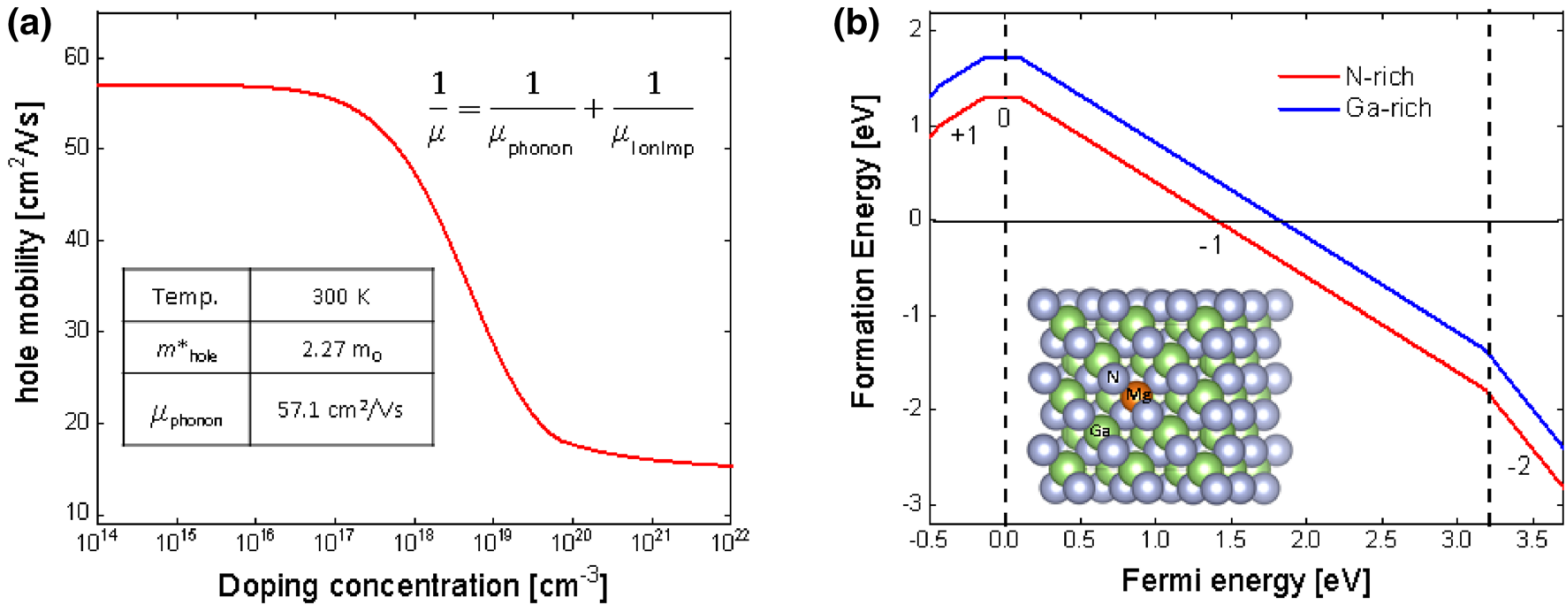
(**a**) Calculated hole mobility as a function of doping concentration in cubic GaN considering both phonons scattering and ionized impurities (IonImp) scattering. The temperature is set at *T* = 300 K. The intrinsic phonon limited mobility, hole effective mass is also listed in the figure. (**b**) The calculated defect formation energy of Mg substitution for Ga as a function of Fermi energy. The zero of Fermi energy is aligned to the valence band edge. A negative Fermi energy means hole degenerate doping condition while Fermi level above conduction band edge means the electron degenerate doping condition. The ionization energy corresponds to the transition level ε(0/− 1) which is about 99.6 meV above the VBM. Two conditions, i.e., N-rich and Ga-rich, are considered. The practical growth condition will fall in between these two extremes. An illustration of the atomic structure of Mg-doped cubic GaN is inserted in the figure.

Figure 1a shows the hole mobility as a function of doping concentration at 300 K for c-GaN. At low doping concentrations in the order of 10^14^ to 10^16^ cm^−3^, the hole mobility is limited by the intrinsic phonon scattering, with an upper limit of 57.1 cm^2^/Vs. The mobility decreases with doping concentration down to a value of 6 cm^2^/Vs for a doping concentration of 10^22^ cm^−3^. Cubic GaN shows a value of hole mobility around 18 cm^2^/Vs at a doping concentration of 5 × 10^19^ cm^−3^, compared to the Hall measurement determined value of ~ 8 cm^2^/Vs (presented in the next sections) for this doping concentration. The difference could be accounted for by other factors, such as surface and interface (c-GaN/GaAs) scattering that were not considered in the present calculation but are expected to exist in real samples. Figure 1b shows the substituting Mg defect formation energy as a function of the Fermi level calculated under both N-rich and Ga-rich conditions. The Ga-rich condition leads to a higher defect formation energy, but the same charge state transition levels when compared with N-rich condition, which is similar to that in hexagonal GaN^34^. The ionization energy level corresponds to the transition level at which the Mg charge state changes from 0 to − 1 (ε(0/− 1)), which is about 99.6 meV above the valence band maximum (VBM) according to our DFT calculation, well consistent with our experimental determined value as will be discussed below. Our further calculation (Supplementary Fig. S1) also reveals an activation energy of 210 meV for Mg in hexagonal phase GaN, suggesting that p-type doping in cubic GaN could be enhanced due to the lower ionization energy in comparison with hexagonal GaN.

### Samples preparation

Cubic GaN layers were grown by plasma-assisted molecular beam epitaxy (PAMBE) on semi-insulating GaAs (001) substrates. A Mg effusion cell was employed at temperatures (T_Mg_) from 360 to 430 °C to produce high values of the Mg flux aiming to obtain heavy p-type doping. The substrate temperature (T_Sub_) was varied between 670 and 700 °C, the N power between 100 and 150 W (with a fixed N flux of 0.4 sccm), depending on the sample (Table S1). The thickness of the samples was measured by cross-section SEM obtaining an average thickness of 400 nm (Fig. S2). The cubic phase structure was confirmed by x-ray diffraction (Fig. S3). Further details on the PAMBE growth and sample characteristics can be found in Supplementary Information.

### Electrical characterization: Hall effect and DLTS analysis

**Figure 2:**
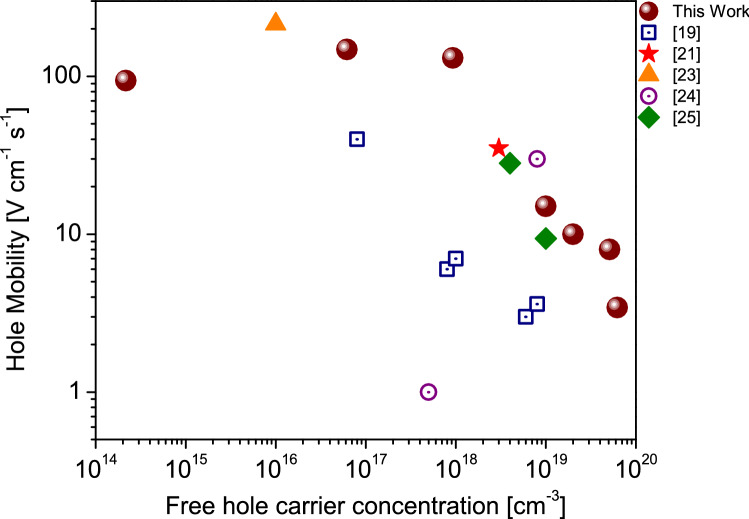
Hall effect mobility as a function of free hole carrier concentration. Our samples are represented by circles and are complemented with previously reported results for Mg-doped c-GaN^19,21,23–25^.

Sample processing and measurement details for the electrical characterization are presented in Supplementary Information. The hole mobility of the samples as a function of hole concentration measured by the Hall effect is shown in Fig. 2. We included in the figure available data reported in the literature for Mg-doped cubic GaN; the hole concentration varies in the order of 10^14^–10^19^ cm^−3^, whilst the mobility in the range of 148–1 cm^2^/Vs. According to our XPS analysis, the samples with a high Nitrogen concentration (fewer Nitrogen vacancies V_N_) presented an increase in hole carrier concentration, in good agreement with reference^35^. The optimized growth conditions were Ga-rich with ζ_N_ / (ζ_Ga_ + ζ_Mg_) ~ 0.9, where ζ_N_, ζ_Ga_, and ζ_Mg_ are the concentration of N, Ga, and Mg, respectively. These growth conditions allowed us to achieve a high free hole carrier concentration in the rage of 6 × 10^19^ cm^−3^ with a mobility value of 3.4 cm^2^/Vs. To the best of our knowledge, the reported free hole concentration in this work is higher than previously reported up to now as is illustrated in Fig. 2. The mobility for samples with high hole concentration is lower than the DFT estimated mobility dominated by ionized impurities scattering, which suggests that other mechanisms, like surface and interface scattering, should be considered. The atomic Mg percentage in the sample with the highest hole concentration is 0.15% (extracted by XPS), which corresponds to a Mg concentration of 1.3 × 10^20^ cm^−3^. Therefore, if the free hole carrier concentration is around 6 × 10^19^ cm^−3^ then the ionization efficiency is 46%. We observed (Fig. S4) in our samples grown with very high Mg fluxes (BEP_Mg_ > 10^–8^ Torr) a strong reduction in hole concentration. Under these growth conditions, Mg exceeds the doping range and its content approximates to the solubility saturation. Thus, Mg atoms might incorporate at places different from the expected Ga sites, and could generate donors causing a self-compensation effect, which would yield to a reduction of hole concentration. Note that a compensating behavior has been observed in hexagonal III-nitrides grown by MBE^36^ and MOCVD^37^. A model involving Mg-V_N_ complex was proposed to explain the self-compensation in heavily Mg-doped p-type h-GaN^38^.

**Figure 3:**
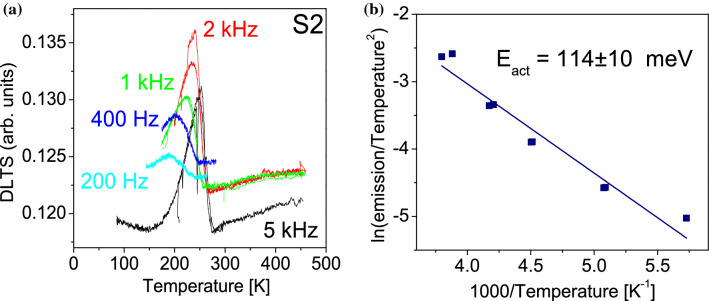
(**a**) DLTS spectra and (**b**) Arrhenius plot of sample S2.

DLTS measurements were performed looking for carrier traps in our samples. The DLTS spectra of sample S2 are presented in Fig. 3, for each repetition rate. We observe a single defect level, the positive peak indicates a minority trap. As the repetition rate diminishes, the DLTS peak moves to lower temperatures, as expected. The emission at each peak maximum coincides with the emission of the deep level, yielding an emission vs absolute temperature dependence. An Arrhenius plot was done, using the standard procedure proposed by Lang^39^, obtaining activation energy of E_act_ = 114 meV. A deep level with an activation energy of 120 meV has been reported in hexagonal GaN, which was extracted from Arrhenius plots of DLTS measurements and assigned to V_N_^40^. For cubic GaN an N-vacancy related defect was obtained by DLTS with an activation energy of 200 meV^41^. These results and the fact that our samples were grown in Ga-rich conditions suggest that the level we observed with an energy E_act_ = 114 meV could be related to N vacancies. Therefore, the reduction in hole concentration for samples grown with BEP_Mg_ > 10^–8^ Torr could be related to a Mg-V_N_ complex, as above mentioned.

### XPS valence band analysis and PL

**Figure. 4:**
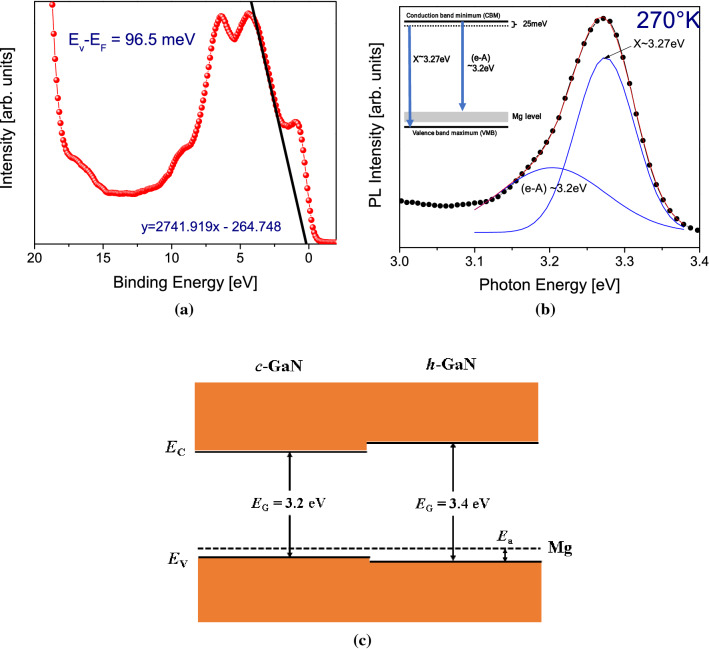
(**a**) Valence band analysis by XPS, (**b**) PL analysis at 270 K, and (**c**) schematic figure showing the band edge positions for c-GaN and h-GaN.

From our DFT calculations, the energy difference between the valence band maximum and the Fermi level is about 99.6 meV. To extract the activation energy on sample S2, we employed the XPS valence band method (XPS-VBM)^42^. In Fig. 4a is observed that the energy difference between the valence band and Fermi level is 96.5 meV, which is very close to that predicted by DFT (99.6 meV). In addition, photoluminescence spectroscopy (PL) measurements were performed to obtain an independent estimation for the Mg acceptor activation energy. The PL measurements were carried out employing a He-Cd Laser and a CCD detector. Figure 4b shows the PL spectrum of the sample S2 at 270 K. Two main transitions were observed corresponding to excitonic transition (X) at 3.27 eV and free electron to the acceptor (e-A) at 3.2 eV, respectively. Therefore, the Mg activation energy is calculated employing the method reported in ref^43^, and correcting the (e-A) peak position by KT/2 (being K the Boltzmann constant and T the absolute temperature)^44^. The energy band diagram extracted by PL analysis is shown in the inset of Fig. 4b. Thus, the acceptor activation energy can be calculated as:

$$E_{Mg} \left( {{\text{eV}}} \right) = E_{x} + \varepsilon_{b}- E_{e{-}A} + KT/2$$

where *E*_*x*_ = 3.27 eV is the c-GaN excitonic transition, ε_*b*_ = 0.025 eV is the free exciton binding energy, *E*_*e-A*_ = 3.2 eV emission is the (e-A) transition, and *KT/2* = 0.011 eV is the temperature correction for (e-A)^44^ . Thus, the Mg ionization energy extracted by PL is 106 meV which is quite close to the predicted by DFT. The low Mg activation energy in c-GaN can be understood by the atomic geometric structure. The in-plane N-Ga bond distance (1.966 Å) in the hexagonal phase is slightly shorter than that in the cubic phase (1.968 Å), which means that the Ga-N bonding interaction in the hexagonal phase is stronger than the cubic phase. In terms of band alignment, the VBM of h-GaN lies lower than that of c-GaN, while the CBM of h-GaN lies higher than that of c-GaN. This also explains why h-GaN has a wider band gap than c-GaN. When both phases are doped with p-type Mg, the ionization energy level of Mg will be located closer to the VBM of c-GaN, as is illustrated in Fig. 4c.

## Conclusions

We have studied the p-type doping in cubic phase GaN under a high flux of Mg atoms. First principles calculation under the DFT formalism was used to predict the transport properties of zincblende GaN and the Mg activation energy. The DFT Mg activation energy was found to be around 99.6 meV. This value matches quite accurately with the experimental results obtained by XPS-VBM and PL. A maximum hole concentration around 6 × 10^19^ cm^−3^ with mobility of 3.4 Vcm^−1^ s^−1^ was achieved. For higher Mg doping a self-compensation effect observed probably caused by Mg-V_N_ complexes.

## Supplementary information

Supplementary file1
