# Supplementary material for: Study of the heavily p-type doping of cubic GaN with Mg
Source: Sci Rep. 2020 Oct 8;10:16858. doi: 10.1038/s41598-020-73872-w (PMC7544912; doi:10.1038/s41598-020-73872-w)
Supplement: Supplementary file 1 — Supplementary file1 [file 41598_2020_73872_MOESM1_ESM.docx]

**SUPPLEMENTARY INFORMATION FOR**

**Study of the heavily p-type doping of cubic GaN with Mg**

C. A. Hernández-Gutiérrez^1,^*, Y. L. Casallas-Moreno^2^, Victor-Tapio Rangel-Kuoppa^3^, Dagoberto Cardona^4^, Yaoqiao Hu^5^, Yuri Kudriatsev^6^, M. A. Zambrano Serrano^7^, Salvador Gallardo Hernandez^8^, and Máximo López López^8,^*

^1^Tecnológico Nacional de México/Instituto Tecnológico de Tuxtla Gutiérrez, Posgrado en Ingeniería grupo de Opto-mecatrónica, Carretera Panamericana km 1080, C.P. 29050, Tuxtla Gutiérrez, México.

^2^CONACYT, Instituto Politécnico Nacional - UPIITA, Av. IPN 2580 Col. Barrio la Laguna Ticomán, Ciudad de México, C.P. 07340, México.

^3^Centro de Investigación en dispositivos semiconductores, Instituto de Ciencias, Universidad Autónoma de Puebla, Puebla, C.P. 72000, México

^4^Facultad de Ciencias Físico-Matemáticas, UMSNH, Edificio L, Francisco J. Mujica s/n, Morelia, Michoacán, C.P. 58000, México

^5^Department of Materials Science and Engineering, The University of Texas at Dallas, Richardson, Texas, C.P. 75080, USA

^6^Department of Electrical Engineering–SEES, Cinvestav-IPN, Ciudad de México, C.P. 07360, México

^7^Nanoscience and Nanotechnology Doctoral Program, Cinvestav-IPN, Ciudad de México, C.P. 07360, México

^8^Physics Department, Cinvestav-IPN, Ciudad de México, C.P. 07360, México

* mlopezl@cinvestav.mx, chernandez@fis.cinvestav.mx

**Figure S1.** The calculated defect formation energy of Mg substitution for Ga as a function of Fermi energy for hexagonal GaN.

**Details of GaN growth**

The Mg-doped cubic GaN layers were grown by PAMBE on semi-insulating GaAs (001) substrates in a Riber C-21 system equipped with standard effusion cells. A radio-frequency (RF) plasma source was used to supply reactive atomic Nitrogen. First GaAs substrates were thermally cleaned at 610 ^o^C for 25 minutes. After this oxide desorption process, a GaAs buffer layer was grown at 0.8 µm/hr, with a substrate temperature of T_Sub_= 595^o^C, a beam equivalent pressure (BEP) of BEP_As_ = 7.2×10^-6^ Torr and BEP_Ga_ = 2.93×10^-7^ Torr, for As and Ga, respectively. GaN layers where grown employing a variety of growth conditions. Nitrogen-rich conditions promoted three-dimensional growth resulting in rough surfaces, therefore we report here results on samples grown in Ga-rich conditions. First, a GaN nucleation layer was grown during 40 s, with a BEP_Ga_ = 2.28×10^-7^ Torr. A series of samples of Mg-doped cubic GaN (c-GaN) were grown, with T_Sub_ between 670 and 700 ^o^C, the Mg effusion cell temperature (T_Mg_) was varied from 360 to 430 ^o^C, and the N power between 100 and 150 W (with a fixed N flux of 0.4 sccm). The value of BEP_Ga_ for Mg-doped c-GaN was the same as for the GaN nucleation layer. The optimized doping growth conditions were Ga-rich with ζ_N_ / (ζ_Ga_ + ζ_Mg_) ~ 0.9, where ζ_N_, ζ_Ga_, and ζ_Mg_ are the concentration of N, Ga and Mg, respectively. Table S1 summaries the growth parameters and characteristics of samples. All c-GaN layers were grown at a 0.3 ML/s. The thickness of the samples was measured by cross-section SEM obtaining an average thickness of 400 nm suitable for hall effect bulk measurements (Fig. S2).

Table S1. Summary of growth conditions and sample characteristics.

| **Sample** | **Mg Cell** T(°C) | **Mg Flux** (Torr) | **T_Substrate_**  (°C) | **N Plasma Power** (W) | XPS  **Ga** (%) | XPS  **N** (%) | XPS  **Mg** (%) | **Hole concentration** (cm^-3^) | **Mobility**  ($\frac{{cm}^{2}}{Vs}$) |
| --- | --- | --- | --- | --- | --- | --- | --- | --- | --- |
| S1 | 355 | 3.0×10^-9^ | 700 | 100 | - | - | - | 1x10^19^ | 15 |
| S2 | 360 | 3.3×10^-9^ | 700 | 100 | 52.8 | 47.1 | 0.1 | 2×10^19^ | 10 |
| S3 | 370 | 5.0×10^-9^ | 700 | 100 | - | - | - | 5.1×10^19^ | 8 |
| S4 | 380 | 8.4×10^-9^ | 700 | 100 | 53.05 | 46.8 | 0.15 | 6.22×10^19^ | 3.43 |
| S5 | 400 | 1.5×10^-8^ | 675 | 150 | - | - | - | 9.27×10^17^ | 131 |
| S6 | 430 | 3.24×10^-8^ | 700 | 150 | 50.7 | 45.9 | 3.4 | 2.16×10^14^ | 94 |
| S7 | 430 | 3.24×10^-8^ | 670 | 150 | - | - | - | 6.2×10^16^ | 148 |

**Figure S2.** Cross-section SEM image of sample S2.


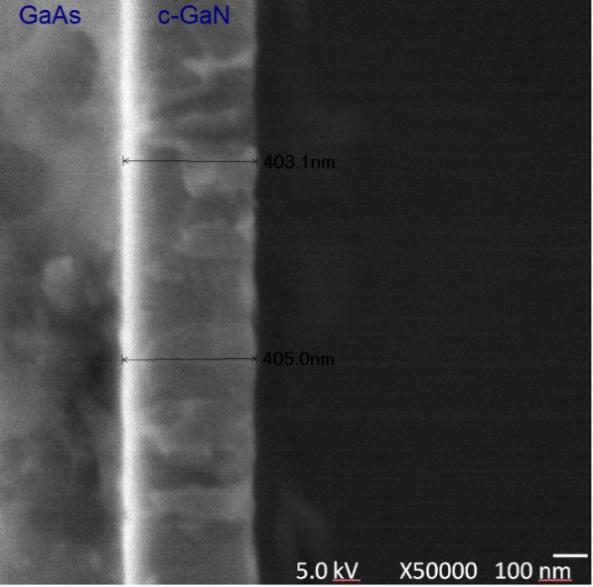


**Figure S3.** X-ray diffraction of samples in the Bragg-Brentano configuration.

**Figure S4.** Variation of hole concentration as a function of Mg beam flux equivalent pressure (BEP_Mg_).

**Sample preparation for the electrical characterization**

The samples were cleaved to achieve an area of 1 cm^2^. For Hall studies, four ohmic contacts were deposited in a van der Pauw geometry. First, the surface was chemically cleaned by HCl and then Ni/Au metals layers were deposited using an e-beam system. Rapid thermal annealing was done next at 490 °C for 8 minutes under a mix of Nitrogen and Oxygen atmosphere. The Ohmic contacts were evaluated by the transmission line method obtaining a specific contact resistance in the range of 3x10^-2^ Ω×cm^2^. Hall measurements were performed employing a current of 0.1 mA and a magnetic field of 5500 Gauss, at room temperature and in darkness.

As for DLTS measurements, since the substrate is semi-insulating, ohmic and Schottky contacts were performed on the c-GaN epilayer. First, the ohmic contact was processed on the epilayer, leaving a well-defined empty hole (i.e., with no metal deposited on it) with a 0.6 mm diameter for the Schottky contacts. A deposition of 50 nm Ni and 50 nm Au annealed at 420 ^o^C for 5 min was used for the ohmic contacts. After annealing, a Schottky contact was done on the empty hole, with a diameter of 0.4 mm, depositing 50 nm Ti and 50 nm Au, separated from the ohmic contacts by a 0.1 mm distance. The electrical circuit of this structure consists of a set of parallel capacitances, on one side connected, and on the other side connected *via* a resistance. As it has been done in former studies with ohmic and Schottky contacts on the epilayer, the DLTS experiments should be done with 0 V to avoid horizontal electric fields that drag charge of the depletion region, hindering thermal emission. At the same time, this brings the following experimental benefits: (i) DLTS scans are not affected by any leakage current, (ii) DLTS measurements are not distorted by a large value of the series resistance (iii) no DLTS signal appears from the interface epilayer/substrate, as the depletion region never reaches the interface. Thus, DLTS studies were done on this structure, which was measured at 0 V, the depletion region was populated with a forward bias of +1 V during 1.3 ms, and DLTS spectra were measured with repetition rates of 5, 2, 1, 0.4 and 0.2 kHz.
